# 454 Pyrosequencing Analysis on Faecal Samples from a Randomized DBPC Trial of Colicky Infants Treated with *Lactobacillus reuteri* DSM 17938

**DOI:** 10.1371/journal.pone.0056710

**Published:** 2013-02-28

**Authors:** Stefan Roos, Johan Dicksved, Valentina Tarasco, Emanuela Locatelli, Fulvio Ricceri, Ulf Grandin, Francesco Savino

**Affiliations:** 1 Department of Microbiology, Swedish University of Agricultural Sciences, Uppsala, Sweden; 2 Department of Pediatrics, Regina Margherita Children Hospital, University of Turin, Torino, Italy; 3 Human Genetics Foundation–Molecular and Genetic Epidemiology Unit, Torino, Italy; 4 Department of Animal Nutrition and Management, Swedish University of Agricultural Sciences, Uppsala, Sweden; 5 Department of Aquatic Sciences and Assessment, Swedish University of Agricultural Sciences, Uppsala, Sweden; University of Glasgow, United Kingdom

## Abstract

**Objective:**

To analyze the global microbial composition, using large-scale DNA sequencing of 16 S rRNA genes, in faecal samples from colicky infants given *L. reuteri* DSM 17938 or placebo.

**Methods:**

Twenty-nine colicky infants (age 10–60 days) were enrolled and randomly assigned to receive either *Lactobacillus reuteri* (10^8^ cfu) or a placebo once daily for 21 days. Responders were defined as subjects with a decrease of 50% in daily crying time at day 21 compared with the starting point. The microbiota of faecal samples from day 1 and 21 were analyzed using 454 pyrosequencing. The primers: Bakt_341F and Bakt_805R, complemented with 454 adapters and sample specific barcodes were used for PCR amplification of the 16 S rRNA genes. The structure of the data was explored by using permutational multivariate analysis of variance and effects of different variables were visualized with ordination analysis.

**Results:**

The infants’ faecal microbiota were composed of *Proteobacteria*, *Firmicutes*, *Actinobacteria* and *Bacteroidetes* as the four main phyla. The composition of the microbiota in infants with colic had very high inter-individual variability with *Firmicutes*/*Bacteroidetes* ratios varying from 4000 to 0.025. On an individual basis, the microbiota was, however, relatively stable over time. Treatment with *L. reuteri* DSM 17938 did not change the global composition of the microbiota, but when comparing responders with non-responders the group responders had an increased relative abundance of the phyla *Bacteroidetes* and genus *Bacteroides* at day 21 compared with day 0. Furthermore, the phyla composition of the infants at day 21 could be divided into three enterotype groups, dominated by *Firmicutes*, *Bacteroidetes*, and *Actinobacteria*, respectively.

**Conclusion:**

*L. reuteri* DSM 17938 did not affect the global composition of the microbiota. However, the increase of *Bacteroidetes* in the responder infants indicated that a decrease in colicky symptoms was linked to changes of the microbiota.

**Trial Registration:**

ClinicalTrials.gov NCT00893711

## Introduction

Infantile colic is one of the most common problems in paediatric medicine. It is a clinical condition characterized by inconsolable crying, fussing and irritability in otherwise healthy newborn infants during the first 3 months of life. The diagnosis is clinical according to an old definition based on crying time, known as the “Wessel’s rule of three”: unexplained episodes of paroxysmal crying for more than 3 h/day for 3 days/week for at least 3 weeks [1]. A recent study identified some perinatal predisposing factors including maternal education, smoking habits, cheese consumption, hostility scores and domestic violence [2]. Despite, however, many studies conducted to determine the cause of this condition, its pathogenesis remains unclear and is most likely multifactorial [3], [4].

Increasing importance has been recently assigned to the role of an aberrant gut microbiota in infants suffering from infantile colic, suggesting its influence on gut motor function and gas production [5] and emphasizing the possible inflammatory origin of this condition [6]. Anaerobic Gram-negative bacteria producing hydrogen gas have been more frequently found in colicky infants, as well as lower numbers and specific colonization patterns of intestinal lactobacilli [7]. Coliform bacteria, particularly *Escherichia coli*, were found to be more abundant in the faeces of colicky infants, suggesting a role for colonic fermentation and consequent excessive intra-intestinal air production, aerophagia and pain, typical in these patients [5], [8]. Further support for a role of the gut microbiota can be found in a study by Rhoads et al. [6], in which elevated levels of fecal calprotectin and a higher level of *Klebsiella* were demonstrated in crying infants, as well as in Pärtty et al. [9] where certain bifidobacteria were inversely associated with colicky symptoms.

Probiotics are live micro-organisms which when administered in adequate amounts confer a health benefit on the host. Recent clinical work demonstrated that administration of probiotic *Lactobacillus reuteri* improved colic symptoms in breastfed infants [10]–[12]. The probiotic may elicit its effect either through direct interaction with the host, or indirectly by affecting the host gastrointestinal microbiota and thereby the colicky symptoms. In fact, it was earlier shown that intake of *L. reuteri* resulted in decreased faecal counts of *E. coli* and an increase of lactobacilli [10].

Recent years have seen a revolution in methodology for the analysis of the composition of bacterial ecosystems. High throughput sequencing of 16 S rRNA genes PCR amplified from the bacteria present in the ecosystem allows mapping of the dominant microbiota, phylogenetic classification of the microbes and comparisons of microbial composition at different phylogenetic levels [13]–[16]. The current study was performed to better understand the complex microbial communities of the colicky infant gut and the effect of supplementation with *L. reuteri*, known to improve colic symptoms, on the global microbiota. Crying patterns of infants were recorded and global faecal microbial composition was analysed at baseline and after 21 days of supplementation, using large-scale DNA sequencing of 16 S rRNA genes. The infants had a remarkably large inter-individual compositional variability and no correlation could be found with treatment. Thus, the probiotic *L. reuteri* DSM 17938 was shown to not affect the global composition of the microbiota. However, there was an increase of *Bacteroidetes* in the group responder infants, irrespective of treatment, indicating that a decrease in colicky symptoms was linked to changes of the global microbial composition.

## Materials and Methods

The protocol for this trial and supporting CONSORT checklist are available as supporting information; see Checklist S1 and Protocol S1.

### Subjects and Methods

We have earlier shown that *Lactobacillus reuteri* DSM 17938 relieves infantile colic [10], [11]. The aim of this study was to evaluate the effect of administration of this bacterium on gut microbiota of the colicky infants and the analyses were done on samples collected during the study by Savino et al. [10]. This randomized, controlled, double-blind study has been performed on 50 breastfed infants consecutively recruited from general pediatricians and from outpatients at the Department of Pediatrics, University of Turin (Regina Margherita Children's Hospital) between March 2008 and August 2009 and in accordance with good clinical practice and the Declaration of Helsinki. All infants had a diagnosis of infantile colic according to Wessel’s criteria: episodes of crying lasting more than 3 h a day, for 3 or more days in the 1 week prior to enrolment. They were all born at term, adequate for gestational age, with a birth weight between 2500 and 4000 g, and were aged 10–60 days at recruitment. Only exclusively breastfed infants were enrolled in order to prevent variability in the intestinal microbiota caused by diet. Exclusion criteria were clinical evidence of chronic illness or gastrointestinal disorders, any intake of probiotics and/or antibiotics in the week preceding recruitment and mixed breast- and formula-feeding.

The sample size was calculated post-hoc to find a clinically relevant difference in the reduction in daily average crying time of 50% between day 1 and day 21. With α = .05, β = .40, 13 patients were needed per group.

For the specific analysis of the faecal microbiota, we evaluated 29 infants. The protocol was approved by the Ethics Committee of the Azienda Ospedaliera, OIRM S. Anna – Ospedale Mauriziano (Turin, Italy). Characteristics of enrolled subjects are reported in Table 1, and no differences were found between the two groups of subjects, besides the response to the treatment. Thus, the previous reported effect of *L. reuteri* DSM 17938 [10] was evident also in this subgroup of subjects. A written informed consent was obtained from both parents before the inclusion of infants. At enrolment, each infant underwent a medical examination and parents completed a questionnaire to obtain data about subjects, including also type of delivery (vaginally or caesarean). Infants were recruited and randomly assigned to receive the probiotic *L. reuteri* DSM 17938 or placebo daily for 21 days. Patients in each of the two groups were defined as “responders” or “non responders”, a responder displaying a reduction of 50% of crying time on day 21. Faecal samples (10–15 g) were collected from each subject, directly from the diaper or anus, at enrolment and on day 21. Samples (blinded) were immediately placed at −20°C and then later stored at −70°C until analysis of the gut microbiota by 454 pyrosequencing.

**Table 1:** Characteristics of subjects.

Variable	Placebo *(n = 14)*	*L. reuteri (n = 15)*	*P* value
Type of delivery (caesarean), n (%)	3 (21.4)	6 (40)	0.427#
Male, n (%)	7 (50)	9 (60)	0.715#
Age at entry (days), median (SD)	29.6 (12.9)	29.8 (11.7)	0.960^§^
Responders, n (%)	7 (50)	14 (93)	0.013^#^ *

# Fisher’s Exact Test; § Wilcoxon Rank Sum Test;

* Significant difference.

### DNA Extraction, PCRs and 454 Pyrosequencing

DNA was extracted from 250 mg of faeces using the MoBio Power Soil DNA Kit (Solana Beach, CA, USA) according to the manufacturer’s instruction with the modification that the bead-beating step was performed 2×45 s, at level 5 on a FastPrep^®^−24 device (MP Biomedicals, Solon, OH, USA).

The 16S rRNA genes were PCR amplified using broad range bacterial primers. The primers: Bakt_341F (CCTACGGGNGGCWGGAG) and Bakt_805R (GACTACHVGGGTATCTAATCC) were complemented with 454 adapters and sample specific barcodes [13]. DNA was amplified with PCR (DreamTaq™ Mastermix, Thermo Fisher Scientific, Fermentas GmbH, St. Leon-Rot, Germany) under the following running conditions: initial denaturation at 95°C for 5 min, 30 cycles of 40 s at 95°C, 40s at 58°C, 1 min at 72°C, and a final elongation step for 7 min at 72°C. PCR products were confirmed using agarose gel electrophoresis and these were subsequently isolated from the gel and purified using GeneJET™ gel extraction kit (Thermo Fisher Scientific). Purified products were quantified using a NanoVue spectrophotometer (GE HealthCare, Uppsala, Sweden) and mixed into equimolar amounts. The mixed pool of PCR products was sequenced from the reverse primer direction at the Swedish Institute for Infectious Disease Control in Solna, using the Roche/454 GS Titanium technology platform (Branford, CT, USA).

### Taxonomic Analysis

The sequence processing was mainly performed as described in Herlemann et al. [13]. In brief, sequences were checked for quality and those that were less than 300 bp in length excluding the primer sequence, contained incorrect primer sequences, or contained any ambiguous base were discarded. Remaining sequences were then subjected to complete linkage clustering using the pyrosequencing pipeline at RDP [17] with a conservative 5% dissimilarity to define OTUs. The most abundant sequence from each OTU was selected as a representative sequence and was taxonomically classified by BLAST searching against a local BLAST database comprised of 600, 316 bacterial 16 S rRNA gene sequences longer than 1,200 bp with good Pintail scores from RDP v. 10.7. The OTU inherited the taxonomy (down to genus level) of the best scoring RDP hit fulfilling the criteria of ≥95% identity over an alignment of length ≥280 bp. Assignment of sequences to samples was based on the 5-bp barcode.

### Statistical Analyses

Comparisons of characteristics of subjects were made using Fisher’s exact test and Wilcoxon rank sum test (SAS V9.2). Changes in microbial community composition during the intervention, for treatment type, response and type of birth, and interactions of these factors were tested using the “adonis” function in the vegan package for R [18]. Adonis is a permutational multivariate analysis of variance using a distance matrix as response variable. We used Euclidean distance and 999 permutations. The change of composition was also tested using principal component analysis (PCA) on a matrix on the difference in abundance of the different phyla or genus after and before treatment, using the vegan package [18]. Confidence ellipses around the centroids of the result categories were used to assess the differentiation of responders and non-responders [18]. In all multivariate analyses, data was transformed by taking the arcsine of the square root of the proportional abundance. Comparison of specific changes in relative abundance during the intervention period was done with a Mann Whitney test using Past [19] (http://folk.uio.no/ohammer/past/). We also explored the structure of the data from the two time points with PCA using Canoco 4.5 and the validity of clustering patterns was confirmed with a distance based MANOVA (Bray Curtis distance; Past software).

## Results and Discussion

The 454 pyrosequencing generated in total 412 000 sequences and the number of sequences per sample were between 1593–21322 (average 6538, median 5997). Phylogenetic classification revealed that the prevalence of the four major phyla varied considerably between the samples (Table 2). All four were found at abundances varying from less than 2% to more than 50%, indicating that none of the phyla was discriminated by the method.

**Table 2:** Relative abundance of the main four bacterial phyla.

Phyla	Mean	Median	Max	Min
*Proteobacteria*	15.4%	12.1%	52.0%	0.04%
*Actinobacteria*	30.2%	30.3%	91.4%	0.1%
*Firmicutes*	37.7%	31.7%	97.4%	1.9%
*Bacteroidetes*	16.3%	0.3%	78.6%	Not detected

The infants’ faecal microbiota were composed of *Proteobacteria*, *Firmicutes*, *Actinobacteria* and *Bacteroidetes* as the four main phyla (Figure S1). The microbiota compositions were shown to have very high inter-individual variability with *Firmicutes*/*Bacteroidetes* ratios varying from 0.025 to 4000 in line with previously published studies on gut microbiota establishment in neonates [20], [21] . On an individual basis, the microbiota was, however, for most individuals relatively stable over time (Figure S1). Although the distribution between the phyla differs slightly from the present investigation, other studies have shown that composition of the microbial communities varies widely from baby to baby [20], [22], but also that distinct structures of each individuals microbial community were noticeable [20].

Treatment with *L. reuteri* DSM 17938 did not affect the global composition of the microbiota (Table 3). However, when comparing responders (>50% reduction in crying time; n = 21; 14 in the *L. reuteri* group and 7 in placebo) with non-responders (n = 8; 1 in the *L. reuteri* group and 7 in placebo) the change of composition at the phyla and genus levels differed between the groups (Table 3). Ordination analyses revealed that this difference depended on an increase of the phylum *Bacteroidetes* (Figure S2) and genus *Bacteroides* (data not shown) during the intervention in the group of responders. Although some individuals only had a small change and the variation was large the difference was significant (p<0.01) and the abundance of *Bacteroidetes* increased on average with 8% in responders but decreased with 18% in non-responders (Figure S3). This is the first time that a high abundance of *Bacteroidetes* has been linked to a decrease in colicky symptoms, although increased diversity and relative abundance of *Bacteroidetes* and *Bacteroides* have been shown to correlate with absence of IgE-associated eczema [22] and this is in line with a reported correlation between eczema and colic [23]. Those groups of bacteria are also indicators of a gastrointestinal microbial ecosystem that has maturated and participates in the breakdown of complex plant polysaccharides [24], which could improve a poor digestion. Furthermore, in a study on patients with severe systemic inflammatory response syndrome, Shimizu et al. [25] reported that gastrointestinal dysmotility was associated with altered gut microbiota, and faecal analysis showed that patients with feeding intolerance had significantly lower numbers of total obligate anaerobes including *Bacteroidaceae* than those in patients without feeding intolerance.

**Table 3:** Pseudo F-ratios from tests using “adonis” of effects in microbial community composition between treatment group, response of treatment, and type of delivery, on a matrix on the difference in abundance before and after treatment.

	Phyla	Taxa	OTU
Treatment	1.02	0.81	0.99
Response	4.09*	2.49*	1.30
Delivery	0.89	0.82	0.99
Treatment×Responders	2.51	0.92	1.18
Treatment×Delivery	0.56	0.44	0.51
Responders×Delivery	3.17	3.66.	2.22

The analyses were performed on three different taxonomic resolutions: Phyla, Taxa and OTU.

.p<0.1, *p<0.05.

A principal component analysis on the microbial composition on day 21 revealed a pattern where three types of microbial compositions (enterotypes) could be seen (Figure S4). This clustering pattern was also evident when samples from day 1 were included in the ordination analysis (data not shown). The validity of the clustering pattern was confirmed with a distance based MANOVA (Bray Curtis distance; p<0.00001) In one type (**A**; n = 12), the relative abundance of *Actinobacteria* was over 40% and *Bacteroidetes* below 1% (11 of 12); in the second type (**B**; n = 9), the relative abundance of *Bacteroidetes* was above 25%; and in the third type (**F**; n = 8), the relative abundance of *Firmicutes* was above 45%, *Actinobacteria* below 2% and *Bacteroidetes* below 1% (7 of 8). Notably, 8 of the 9 type B samples, with a high abundance of *Bacteroidetes*, came from the group responders, and 7 of those came from infants with spontaneous delivery (Figure S4). The existence of different host-microbial symbiotic states in adults was recently described [14] and it is intriguing to see “enterotype-like” clustering in colicky infants and that this correlates to the improvement in colic symptoms to some extent.

Vaginally delivery of the infants was overrepresented in enterotype group B and several other studies have reported that method of delivery influenced the microbiota in the first period of life. Caesarean section has been shown to delay colonization by *Escherichia coli, Bacteroides* and *Bifidobacterium* [26] and a negative correlation between *Bacteroides* and cesarean section has been described [27]. Recently Jost et al. [28] performed a study, using pyrosequencing, to investigate the establishment of pioneer gut microbiota during the first month of life of vaginally and exclusively breastfed infants. An early onset of *Bacteroides* was observed in 4 of 7 subjects, demonstrating that those strict anaerobes can reach high densities already during the first weeks of life. In this regards conflicting findings have been published previously [29].

Our current knowledge of the intestinal microbiota and its role in infant colic is weak. The question is if infantile colic is a cause or consequence of an alteration in gut microbiota. Illustrating the complexity of the interaction, the gut microbiota has been described to be involved in the regulation of various host metabolic pathways, giving rise to interactive host-microbiota metabolic, signaling, and immune-inflammatory axes that physiologically link the gut to the brain [30]. The change of the composition of the microbiota detected in the responders could not be attributed to treatment with *L. reuteri*. Consequently, the mechanisms by which this bacterium reduces colic symptoms may be either by modulating of the microbiota in a way that is not detected by 454 pyrosequencing, or by a direct effect on the infant. Preidis et al. [31] reported that *L. reuteri* DSM 17938, but not an other *L. reuteri* strain, considerably increased enterocyte migration, proliferation and crypt height in an experimental model on neonatal mice. The possible role of these mechanisms in decreasing colic symptoms is not known, but they may represent novel mechanisms for *L. reuteri* strain-specific effects. Although no effect on the microbiota could be attributed to supplementation with *L. reuteri* DSM 17938, the results of the study may provide the basis for further investigation of the role of the intestinal microbiota in infants with colic.

## Supporting Information

Figure S1 **Bacterial composition of 29 infants at the phylum level.** The first column in each pair shows the initial composition and the second the composition at day 21. The different phyla are indicated by different colors and the infants responding to the treatment as well as the individuals given *L. reuteri* are marked in the figure.
(EPS)

Figure S2 **Principal component analysis on the difference in abundance of different phyla before and after treatment.** Ellipses show 95% confidence around the centroids of the two outcomes of the treatment. Filled circles are responders and empty non-responders.
(EPS)

Figure S3 **Change in relative abundance of** ***Bacteroidetes*** **during the intervention period.** The group of responders had an increase of this bacterial phylum and differed from the group of non-responders (*P* = 0.004; Mann Whitney test).
(EPS)

Figure S4 **PCA plot from faecal samples on day 21.** Bacterial phyla indicated in circles: black, *Actinobacteria*; red, *Bacteroidetes*; blue, *Proteobacteria*, and green, *Firmicutes*. The small inlaid figure describes the features of the samples: purple, responders; green, non-responders; triangles, spontaneous delivery; and squares, caesarean delivery.
(EPS)

Checklist S1 **The CONSORT 2010 checklist shows all the information about the randomised trial.**
(DOC)

Protocol S1 **Trial Protocol.**
(DOC)
