# Supplementary material for: 454 Pyrosequencing Analysis on Faecal Samples from a Randomized DBPC Trial of Colicky Infants Treated with Lactobacillus reuteri DSM 17938
Source: PLoS One. 2013 Feb 28;8(2):e56710. doi: 10.1371/journal.pone.0056710 (PMC3585302; doi:10.1371/journal.pone.0056710)
Supplement: Protocol S1 — Trial Protocol. (DOC) [file pone.0056710.s006.doc]

**STUDY PROTOCOL**

**TITLE**

**Lactobacillus Reuteri *versus* Placebo in the treatment of Infantile Colic: a clinical randomized BBPC study and microbiological study.**

*Translation for the submission to Plos One*

**Version - October 2007**

**Principal Investigator:**

Francesco Savino MD, PhD

Dipartimento di Scienze Pediatriche e dell’Adolescenza

Ospedale Infantile Regina Margherita

Piazza Polonia 94 - 10126 Torino - ITALY

Tel: +390113135257

Fax: +39011677082

E-mail: [francesco.savino@unito.it](mailto:francesco.savino@unito.it)

**BACKGROUND**

Infantile colic is one of the most common problems within the first three months of life. It consists of a behavioural syndrome characterized by paroxysmal, excessive and inconsolable crying without identifiable cause. Although infantile colic is commonly reported and causes appreciable distress for both parents and paediatricians, despite forty years of research, its aetiology still remains unclear. Recently the role of intestinal microflora has growing importance, and a lower count of intestinal lactobacilli has been observed in colicky infants compared to healthy ones. In a recent study we have observed that Lactobacillus reuteri improved colicky symptoms in breast-fed infants within one week of treatment compared to Simethicone, suggesting that probiotics may play a role in infantile colic (Savino F, Pelle E, Palumeri E, Oggero R, Miniero R. *Lactobacillus reuteri* (American Type Culture Collection Strain 55730) versus simethicone in the treatment of infantile colic: a prospective randomized study. Pediatrics 2007; 119:e124-30). The mechanism involved in such finding is only partially understood and, for this reason, we have planned this new study to evaluate not only the clinical improvement but also the microbiological data.

**AIM**

The aims of this study are:

- to evaluate the improvement of colicky symptoms by the oral administration of *L. reuteri:*

-primary outcome: reduction of the daily average crying time from baseline to the end of the treatment period, to less than 3 hours a day, the cut-off proposed by Wessel;

- secondary outcome: number of responders versus non-responders in each group on day 21. Responders (defined in the protocol) were those who experienced a decrease in the daily average crying time of 50% from baseline).

- to study the intestinal microflora of colicky infants before and after *L. reuteri* or placebo administration, evaluating the effect of *L. reuteri* on the growth of the main intestinal microbiota: using fluorescent in situ hybridization (FISH) (coliforms, *Clostridium butyricum*, Lactobacilli, Bifidobacteria). Further, the global intestinal microflora composition, using large-scale DNA sequencing of 16S rRNA genes ( 454-pyrosequencing technique)

**STUDY DESIGN**

***Subjects and methods***

We will enroll full-term, healthy, exclusively breast-fed infants admitted at the Department of Paediatrics and Adolescence, Regina Margherita Children Hospital, Turin, Italy starting September 2008 and in accordance with good clinical practice and the Declaration of Helsinki. A written informed consent will be obtained from both parents before the inclusion of infants.

Inclusion criteria are:

- clinical diagnosis of infantile colic according to Wessel's criteria
- gestational age between 37 and 42 weeks
- age between 4 and 16 weeks
- birth weight between 2500 and 4000 g
- exclusively breastfed.

Exclusion Criteria are:

- clinical evidence of chronic illness or gastrointestinal disorders
- administration of probiotics and antibiotics.

In this prospective, randomized, double blind, placebo-controlled study eligible subjects will be randomized to receive *L. reuteri* DSM 17938 (L) – other name Reuterin BIOGAIA (administered at a dose of 1.000.000.000 colony forming units - CFU) or placebo (P) – other name Placebo BIOGAIA. The paediatrician allocates the next available product on entry into the trial, and each patient received study product directly from the Department. Active study product consists of a suspension of freeze-dried *L. reuteri* DSM 17938 in a mixture of sunflower oil and a medium-chain triglyceride oil supplied in a 5 ml dark bottle fitted with a dropper cap. Placebo is identical in appearance and taste but without the live bacteria. Both formulations will be administered in 5 drops, once a day, thirty minutes before the feed in the morning, for 21 days. During the study, parents will be instructed to refrigerate the product when it was not in use. The bottles will be coded and blinded by the study statistician for both the participants and for the physicians and the code will be revealed to the investigators once recruitment, data collection and all laboratory and statistical analyses will be complete.

Randomization will be performed by the random-digit method, on the basis of a computer-generated numbers. We use a two treatment randomization scheme with random block of varying size (Stata Statistical Software: Release 9.2 StataCorp LP, College Station, TX. Ralloc Procedure). The computer-generated randomization list will be created by an independent departmental statistician (RC or FR).

At enrollment (day 0), parents will be interviewed about gestational age, type of delivery, birth weight, presence of gastrointestinal disease, and crying time. Medical examinations will be performed and growth parameters recorded at baseline and day 21. Parents will be asked to fill in a structured diary to record the daily crying time (minutes), stool characteristics and frequency and any side effects observed (constipation, vomiting and cutaneous reactions) on each day of the study. To assess tolerance, growth parameters (weight gain per day), stooling characteristics (frequency and consistency), symptoms of digestive intolerance (constipation, regurgitation or vomiting) and frequency of adverse events during the treatment period, will be evaluated. Adverse events, defined as illnesses, signs or symptoms that occurred or got worse during the course of the study, will be assessed through parental interview on their daily records. A general linear model for repeated measures will be used to assess differences in growth parameters between groups at the different time points (day 0-21). Follow-up visits will be performed by the same pediatrician on day 7 and 21. ( see FLOW-CHART)

***Statistical analyses***

Data will be described using median and interquartile range or number and percentage for quantitative or qualitative variables, respectively. Comparisons will be made using Fisher’s exact test, Wilcoxon rank-sum test and Mann Whitney test. We will explore the structure of our data using a principal component analysis (PCA). Significance was reached with *P* < 0.01.

The analyses were performed using SAS V9.2 and Past.

Sample size was calculated to find a clinically relevant difference in the reduction in daily average crying time of 50 minutes between groups. With α= .05, β= .20 and an estimated standard deviation (SD) within groups of 55 minutes, 20 patients were needed per group. Twenty-five subjects per group will be enrolled to allow for a 20% drop out rate.

***Analysis of bacterial groups by FISH***

Intestinal bacterial groups will be enumerated using specific Fluorescence In Situ Hybridization (FISH) commercial kits (Microscreen B.V., Microbial Diagnostics, Groningen, The Netherlands) for the *Lactobacillus* group (Lactobacillus 10-ME-H006), the *Bifidobacterium* genus (Bifidobacterium 10-ME-H001), *Escherichia coli* group (Escherichia 1 coli 10-ME-H004) and *Clostridium butyricum* (Clostridium butyricum 10-ME-H009). Slides will be enumerated in triplicate using a Nikon Eclipse E-600 epifluorescence microscope equipped with a mercury arc lamp (Nikon, HBO, 100 W) and the FITC specific filter Nikon BA 520. Depending on the number of fluorescent cells, 30–100 microscopic fields will be counted and averaged in each slide.

***Analysis of bacterial groups by 454-pyrosequencing***

DNA will be extracted from 250 mg of faeces using the MoBio Power Soil DNA Kit (Solana Beach, CA, USA) according to the manufacturer’s instruction with the modification that the bead-beating step was performed 2 x 45s, at level 5 on a FastPrep®-24 (MP Biomedicals, Solon, OH).

The 16S rRNA genes will be PCR amplified using broad range bacterial primers. The primers: Bakt_341F (CCTACGGGNGGCWGGAG) and Bakt_805R (GACTACHVGGGTATCTAATCC) will be complemented with 454 adapters and sample specific barcodes. DNA will be amplified with PCR (DreamTaq^TM^ Mastermix, Thermo Fisher Scientific, Fermentas GmbH, St. Leon-Rot, Germany) under the following running conditions: Initial denaturation at 95º C for 5 min, 30 cycles of 40s at 95º C, 40s at 58º C, 1 min at 72º C, and a final elongation step for 7 min at 72º C. PCR products will be confirmed using agarose gel electrophoresis and these will be subsequently isolated from the gel and purified using GeneJET^TM^ gel extraction kit (Thermo Fisher Scientific). Purified products will be quantified using a NanoVue spectrophotometer (GE HealthCare, Uppsala, Sweden) and mixed into equimolar amounts. The mixed pool of PCR products will be sequenced from the reverse primer direction at the Swedish Institute for Infectious Disease Control in Solna, using the Roche/454 GS Titanium technology platform (Branford, CT, USA).

***Taxonomic analysis***

Sequences were checked for quality and those that were less than 300 bp in length excluding the primer sequence, contained incorrect primer sequences, or contained any ambiguous base will be discarded. Remaining sequences will be then subjected to complete linkage clustering using the pyrosequencing pipeline at RDP with a conservative 5% dissimilarity to define OTUs. The most abundant sequence from each OTU will be selected as a representative sequence and will be taxonomically classified by BLAST searching against a local BLAST database comprised of 600, 316 bacterial 16S rRNA gene sequences longer than 1,200 bp with good Pintail scores from RDP v. 10.7. The OTU inherited the taxonomy (down to genus level) of the best scoring RDP hit fulfilling the criteria of ≥ 95% identity over an alignment of length ≥ 280 bp. Assignment of sequences to samples will be based on the 5-bp barcode.

***Faecal samples storage and transfer***

Feces samples (10-15g) for microbiological analysis will be collected by the investigators from each subject, directly from the diaper or anus, at enrollment and on day 21. Samples (blinded) will be immediately placed at -20°C and then later stored at -70°C until analysis of the gut microbiota by 454-pyrosequencing.

Faecal samples will be stored in dry-ice in polystyrene boxes and then transferred by using TNT express service.

**References**

1: WESSEL MA, COBB JC, JACKSON EB, HARRIS GS Jr, DETWILER AC. Paroxysmal fussing

in infancy, sometimes called colic. Pediatrics. 1954 Nov;14(5):421-35. PubMed

PMID: 13214956.

2: Savino F, Pelle E, Palumeri E, Oggero R, Miniero R. Lactobacillus reuteri

(American Type Culture Collection Strain 55730) versus simethicone in the

treatment of infantile colic: a prospective randomized study. Pediatrics. 2007

Jan;119(1):e124-30. PubMed PMID: 17200238.

3: Savino F, Castagno E, Bretto R, Brondello C, Palumeri E, Oggero R. A

prospective 10-year study on children who had severe infantile colic. Acta

Paediatr Suppl. 2005 Oct;94(449):129-32. PubMed PMID: 16214780.

4: Savino F, Bailo E, Oggero R, Tullio V, Roana J, Carlone N, Cuffini AM,

Silvestro L. Bacterial counts of intestinal Lactobacillus species in infants with

colic. Pediatr Allergy Immunol. 2005 Feb;16(1):72-5. PubMed PMID: 15693915.

5: Savino F, Cresi F, Pautasso S, Palumeri E, Tullio V, Roana J, Silvestro L,

Oggero R. Intestinal microflora in breastfed colicky and non-colicky infants.

Acta Paediatr. 2004 Jun;93(6):825-9. PubMed PMID: 15244234.
